# Administration of losartan preserves cardiomyocyte size and prevents myocardial dysfunction in tail-suspended mice by inhibiting p47^*phox*^ phosphorylation, NADPH oxidase activation and MuRF1 expression

**DOI:** 10.1186/s12967-019-2021-1

**Published:** 2019-08-22

**Authors:** Liwen Liang, Wenyi Yuan, Lina Qu, Huili Li, Lulu Zhang, Guo-Chang Fan, Tianqing Peng

**Affiliations:** 1 Institutes of Biology and Medical Sciences, Soochow University, Suzhou, 215123 China; 2 State Key Laboratory of Space Medicine Fundamentals and Application, China Astronaut Research and Training Center, Beijing, 100094 China; 3 Department of Pharmacology and Systems Physiology, University of Cincinnati College of Medicine, Cincinnati, OH 45267 USA

**Keywords:** Angiotensin-II, Losartan, Microgravity, MuRF1, NADPH oxidase, p47^*phox*^ phosphorylation, Oxidative stress

## Abstract

**Background:**

Spaceflight or microgravity conditions cause myocardial atrophy and dysfunction, contributing to post-flight orthostatic intolerance. However, the underlying mechanisms remain incompletely understood and preventive approaches are limited. This study investigated whether and how losartan, a blocker of angiotensin-II receptor, preserved cardiomyocyte size and prevented myocardial dysfunction during microgravity.

**Method:**

Adult male mice were suspended with their tails to simulate microgravity. Echocardiography was performed to assess myocardial function. Heart weight and cardiomyocyte size were measured. NADPH oxidase activation was determined by analyzing membrane translocation of its cytosolic subunits including p47^*phox*^, p67^*phox*^ and Rac1. Heart tissues were also assayed for oxidative stress, p47^*phox*^ phosphorylation (Ser345), MuRF1 protein levels and angiotensin-II production.

**Results:**

Tail-suspension for 28 days increased angiotensin-II production in hearts, decreased cardiomyocyte size and heart weight, and induced myocardial dysfunction. Administration of losartan preserved cardiomyocyte size and heart weight, and prevented myocardial dysfunction in tail-suspended mice. These cardioprotective effects of losartan were associated with inhibition of p47^*phox*^ phosphorylation (Ser345), NADPH oxidase and oxidative stress in tail-suspended mouse hearts. Additionally, the NADPH oxidase inhibitor, apocynin, also reduced oxidative stress, preserved cardiomyocyte size and heart weight, and improved myocardial function in tail-suspended mice. Furthermore, losartan but not apocynin attenuated tail-suspension-induced up-regulation of MuRF1 protein in mouse hearts.

**Conclusions:**

Administration of losartan preserves cardiomyocyte size and prevents myocardial dysfunction under microgravity by blocking p47^*phox*^ phosphorylation and NADPH oxidase activation, and by inhibiting MuRF1 expression. Thus, losartan may be a useful drug to prevent microgravity-induced myocardial abnormalities.

## Background

Spaceflight or microgravity conditions induce myocardial abnormalilities including atrophy and functional depression [1–3]. Depressed myocardial function during microgravity contributes to post-flight orthostatic intolerance, which may be detrimental not only to the astronauts themselves but to the success of future long-duration space missions [4, 5]. However, the effective approaches to prevent myocardial abnormalities during microgravity are limited.

It has been reported that the renin–angiotensin-II system is activated in human blood during spaceflight [6] and head-down bed rest [7]. Importantly, angiotensin-II signaling has been implicated in promoting skeletal muscle atrophy [8]. Angiotensin-II exerts its action via at least two distinct receptor subtypes designated angiotensin-II type 1 receptor (AT1R) and type 2 receptor (AT2R) [9]. The AT1R signaling has been well addressed in literature [10]. Upon activation of AT1R, angiotensin-II induces phospholipase C signaling through coupling to G_q/11_ and G_i/o_ thereby increasing the cytosolic Ca^2+^ concentrations and subsequently triggering cellular responses [10]. The well established effect mediated by the AT1R is vasoconstriction and thus blockage of AT1R has been an effective approach to lower blood pressure [11]. In addition, angiotensin-II has direct detrimental effects on cardiomyocytes [11]. However, it is unknown whether blockage of angiotensin-II signaling has any beneficial effects on microgravity-induced myocardial abnormalities.

Activation of angiotensin-II signaling is associated with NADPH oxidase activation [10] and subsequent reactive oxygen species (ROS) production in the heart. NADPH oxidase, with its generically termed NOX isoforms, is one of the major sources of ROS in cardiomyocytes with NOX2 and NOX4 being the most predominant isoforms [12, 13]. The NOX2-containing NADPH oxidase is composed of a membrane-bound complex and a cytosolic complex. The membrane-bound complex consists of NOX2 (or gp91^*phox*^) and p22^*phox*^. The cytosolic complex consists of p40^*phox*^, p47^*phox*^, p67^*phox*^ and a small G protein Rac1. During the resting state, the cytosolic complex is separated from the membrane complex. Upon stimulation, cytosolic subunits (e.g. p47^*phox*^) are phosphorylated and then translocated to the membranes, where cytosolic and membrane-bound complexes assemble into functional NADPH oxidase to produce superoxide anion [12, 14]. Notably, residual phosphorylation of Ser345 on p47^*phox*^ is critically important for full activation of the NADPH oxidase [14]. In contrast, NOX4 is primarily regulated through transcriptional mechanisms [15]. NADPH oxidase-derived ROS has been reported to promote skeletal muscle atrophy in various scenarios [2, 16, 17]. However, it remains to be determined whether NADPH oxidase is activated in the heart during microgravity and if so, whether and how blockage of angiotensin-II signaling inhibits NADPH oxidase activation thereby preventing microgravity-induced myocardial abnormalities.

In this study, we investigated whether and how administration of losartan, a well-known blocker of angiotensin-II receptor, prevents NADPH oxidase activation thereby preserving cardiomyocyte size and improving myocardial function in a mouse model of tail-suspension-simulated microgravity.

## Methods

### Animals

This investigation conforms to the Guide for the Care and Use of Laboratory Animals published by the US National Institutes of Health (NIH Publication, 8th Edition, 2011). Breeding pairs of C57BL/6 mice were purchased from the Jackson Laboratory. A breeding program was implemented at Soochow University’s animal care facilities. All experimental protocols were approved by the Animal Use Subcommittee at the Soochow University, China.

### Experimental protocol

Adult male mice (aged 2 months) were suspended by their tails with tape to simulate microgravity using the method described previously [18]. During the tail-suspension procedure, mice had free access to food and water. A well-known blocker for AT1R, losartan or a selective inhibitor of NADPH oxidase, apocynin was given in drinking water to mice during the tail-suspension. Each mouse consumed approximately 80 mg/kg/day of losartan (Energy Chemical, China) and 30 mg/kg/day of apocynin (Sigma-Aldrich, USA). These doses of losartan [19] and apocynin [20] were chosen based on prior studies. Tail-suspension was maintained for 28 days.

### Echocardiography

Mice were lightly anaesthetized with inhalant isoflurane (0.5–1%) and imaged using a 40-MHz linear array transducer attached to a preclinical ultrasound system (Vevo 2100, FIJIFILM VisualSonics, Canada) with nominal in-plane spatial resolution of 40 μm (axial) × 80 μm (lateral). M-mode and 2-D parasternal short-axis scans (133 frames/second) at the level of the papillary muscles were used to assess changes in left ventricle (LV) end-systolic inner diameter, LV end-diastolic inner diameter, LV posterior wall thickness in end-diastole and end-systole, fractional shortening (FS%) and ejection fraction (EF%).

### Histological analyses

Mice were euthanized by cervical dislocation under anesthesia using a mixture of ketamine (100 mg/kg)/zylaxine (5 mg/kg, i.p.). Heart tissues were routinely collected, fixed, processed and sectioned. For cardiomyocyte size measurement, several cross-sections of the heart (5-μm thick) were prepared and stained for cell membranes and nuclei with fluorescein isothiocyanate–conjugated wheat germ agglutinin (Thermo Fisher Scientific) and Hoechst33342 (Thermo Fisher Scientific), respectively. A single cardiomyocyte was measured by using an image quantitative digital analysis system (NIH Image version 1.6) as previously described [21]. The outlines of at least 200 cardiomyocytes were traced in each section.

### Analysis of differentially expressed genes

mRNA microarray was performed to analyze gene expression in sham and tail-suspended mouse hearts through collaboration with Oebiotech Inc (China). Briefly, total RNA was extracted and purified from heart tissues using the mirVana™ RNA Isolation Kit and QIAGEN RNeasy^®^ Mini Kit, respectively, following the manufacturers’ instructions. cRNA synthesis, labeling, fragmentation and hybridization were sequentially conducted using the Agilent Oligo Microarray Kit. The slides were scanned using the Agilent Microarray Scanner (Agilent p/n G2505C). Data was extracted using the Agilent Feature Extraction Software. The mRNA expression profiles were deposited into the gene expression omnibus (GEO) database (GSM3567530, GSM3567531 and GSM3567532 for sham group, and GSM3567533, GSM3567534 and GSM3567535 for tail-suspension group).

### Measurement of angiotensin**-**II in heart tissues

The levels of angiotensin-II were measured in whole heart lysates using a mouse angiotensin-II (ANG-II) ELISA Kit (CUSABIO, China) following the manufacturer’s instructions.

### Determination of oxidative stress

The formation of ROS in heart tissue lysates was measured using the Amplex^®^ Red Hydrogen Peroxide/Peroxidase Assay Kit (Thermo Fisher Scientific) according to the manufacturer’s instructions. Briefly, frozen heart tissues were homogenized in an assay buffer. The homogenates (50 μg protein) were incubated with a fluorescent probe Amplex^®^ Red and hydrogen peroxide/peroxidase at 37 °C. The fluorescent product was measured using a spectrofluorometer at the 485/525 nm. Changes in fluorescence were expressed as arbitrary units.

Protein carbonyl content was determined using a Protein Carbonyl Colorimetric Assay Kit (Cayman Chemical Company, USA) following the manufacturer’s instructions.

The lipid peroxidation in heart tissue lysates was assessed by measuring malondialdehyde (MDA) production using a TBARS assay kit (Cayman Chemical, USA) following the manufacturer’s instructions.

### Western blot analysis

40 μg of heart tissue lysates or 10 μg of isolated membrane lysates were loaded on sodium dodecyl sulfate polyacrylamide electrophoresis gels. After electrophoresis, the separated proteins were transferred onto Bio-Rad PVDF membranes. The membranes were incubated with antibodies against Rac1 (1:1000 dilution, Abcam), ubiquitin ligase muscle RING-finger protein-1 (MuRF1, 1:1000 dilution, Abcam), Na^+^/K^+^ ATPase (1:1000 dilution, Abcam), p67^*phox*^ (1:1000 dilution, Abcam), residual phosphorylation of Ser345 on p47^*phox*^ and total p47^*phox*^ (1:1000 dilution, Thermo Fisher Scientific Inc.), NOX4 (1:1000 dilution, Abcam) and glyceraldehyde 3-phosphate dehydrogenase (GAPDH, 1:5000 dilution, Cell Signaling Technology), respectively. After washing, the membranes were incubated with relevant secondary antibodies conjugated with horseradish peroxidase. The signals were then developed using an enhanced version of the chemiluminescence reaction.

### Real-time reverse transcription-polymerase chain reaction (RT-PCR)

Real-time RT-PCR was performed to analyze mRNA expression for apelin, apelin receptor and *Gapdh* as previously described [22]. The sequences of primers are as follows: 5′-CCACTGATGTTGCCTCCAGA-3′ (*Apelin* forward primer), 5′- GCGAAATTTCCTCCTGCCTC-3′ (*Apelin* reverse primer), 5′-TTGACCGATACCTGGCCATT-3′ (*Apelin receptor* forward primer), and 5′-GAACACCATGACAGGCACAG-3′ (*Apelin receptor* reverse primer).

### NADPH oxidase activation

NADPH oxidase activation was determined by measuring the translocation of Rac1, p47^*phox*^ and p67^*phox*^ to cell membranes. Briefly, cell membranes were isolated from heart tissues using a commercial kit (Beyotime Biotechnology, Shanghai, China) according to the manufacturer’s instructions. The protein levels of Rac1, p47^*phox*^ and p67^*phox*^ in cell membranes and NOX4 in whole heart lysates were analyzed by western blot. Na^+^/K^+^ ATPase and GAPDH were used as loading controls for cell membranes and whole heart lysates, respectively.

### Caspase-3 activity

Caspase-3 activity in heart tissue lysates was measured using a caspase-3 fluorescence assay kit (Biomol Research Laboratories, USA).

### Superoxide dismutase (SOD) activity

SOD activity in heart tissue lysates was measured using a total SOD assay kit with WST-8 (Beyotime, China).

### Neonatal mouse cardiomyocyte cultures and simulated microgravity

Neonatal mice (born within 24 h) were euthanized by decapitation. Neonatal cardiomyocytes were prepared and cultured according to methods described previously [22].

To simulate microgravity, cardiomyocytes were dispersed in a 5-ml of culture media and then inoculated them through the Syringe Port inside the 5 ml High Aspect Ratio Vessels of the Rotary Cell Culture System (RCCS-4, Synthecon Incorporated, USA). After being assembled, they were placed on their rotary base and maintained in a 37 °C incubator with a 5% CO_2_/air mixture and saturating humidity. Vessel rotation was set at 20 rpm according to a previous report [23]. After 24 h, cells were collected for western blot analysis.

### Statistical analysis

All data were expressed as mean ± SD. Differences between two groups were compared by unpaired Student’s t test. For multi-group comparisons, ANOVA followed by Newman–Keuls test was performed. *P* value < 0.05 was considered statistically significant.

## Results

### Tail-suspension induces an imbalance between angiotensin**-**II and apelin systems in the heart

**Fig. 1:**
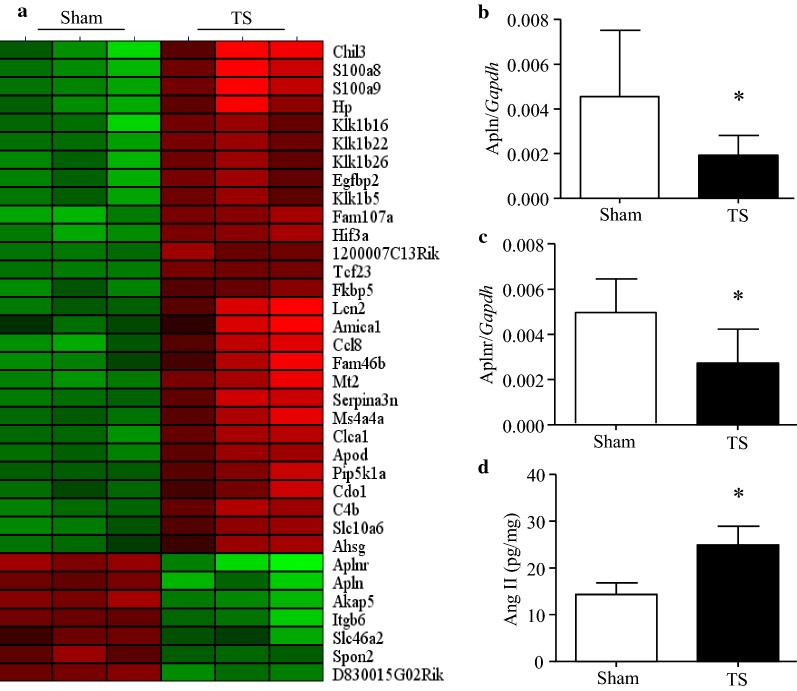
Tail-suspension reduces apelin/apelin receptor expression and increases angiotensin-II production in mouse hearts. After tail-suspension (TS), cDNA microarray was performed to determine differential expression of genes in mouse hearts. **a** Heat map of differential gene expression. **b**, **c** The mRNA levels of apelin and apelin receptor relative to *Gapdh* were analyzed by real-time RT-PCR. **d** Angiotensin-II production was measured in heart lysates. Data are mean ± SD, n = 6 in each group. *P < 0.05

Microarray analysis identified the difference of gene expression between sham and tail-suspended mouse hearts (Fig. 1a). Among differentially expressed genes, apelin and its receptor were the most down-regulated genes in tail-suspended compared with sham mouse hearts (Fig. 1a). Down-regulation of apelin and its receptor in tail-suspended mouse hearts was further confirmed by real-time RT-PCR (Fig. 1b, c). This finding indicated that the apelin/apelin receptor system might be compromised in tail-suspended mouse hearts.

Since the apelin/apelin receptor system counteracts the action of angiotensin-II [24], we measured the protein levels of angiotensin-II in heart tissues. Tail-suspension for 28 days induced an increase in angiotensin-II levels by 73.49% (Fig. 1d). Taken together, these results suggest that tail-suspension may induce an imbalance between angiotensin-II and apelin/apelin receptor system in the heart with the former being increased.

### Administration of losartan improves myocardial function and increases cardiomyocyte size and heart weight in tail-suspended mice

**Fig. 2:**
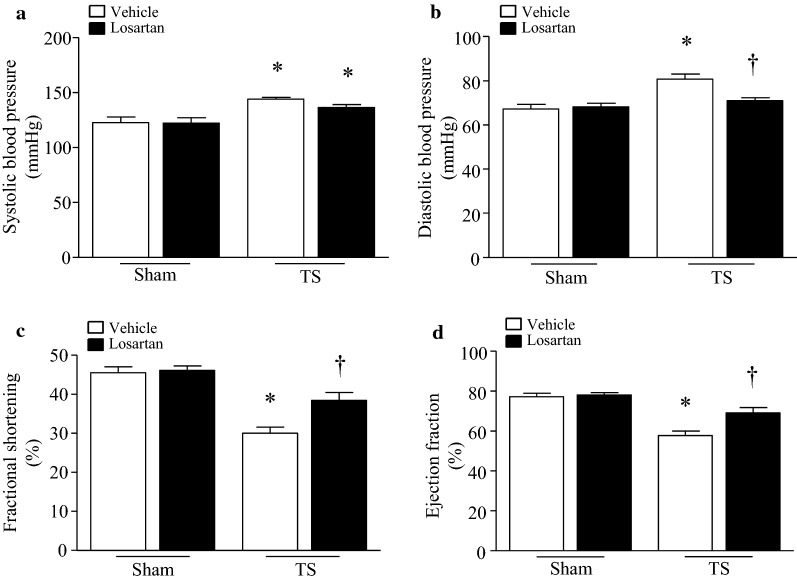
Administration of losartan lowers diastolic blood pressure and attenuates myocardial dysfunction in tail-suspended mice. Tail-suspended mice were given losartan or vehicle in drinking water for 28 days. **a**, **b** Blood pressure was measured in mice. **c**, **d** Ejection fraction (EF)% and fraction shortening (FS)%. Data are mean ± SD, n = 6 in each group. *P < 0.05 vs. Sham + Vehicle and ^†^*P* < 0.05 vs. TS + Vehicle

Having shown that angiotensin-II was elevated and apelin/apelin receptor decreased in the heart, we hypothesized that increased angiotensin-II contributes to myocardial abnormalities during tail-suspension. To address this hypothesis, we gave losartan in drinking water to mice, a pharmacological blocker for AT1R clinically used to lower blood pressure. Administration of losartan lowered diastolic blood pressure in tail-suspended mice but not sham animals (Fig. 2a, b). Tail-suspension for 28 days resulted in myocardial depression as determined by decreased EF% and FS% (Fig. 2c–f). However, EF% and FS% were much greater in losartan-treated mice compared with vehicle-treated group after tail-suspension. Administration of losartan did not affect myocardial function in sham animals.

**Fig. 3:**
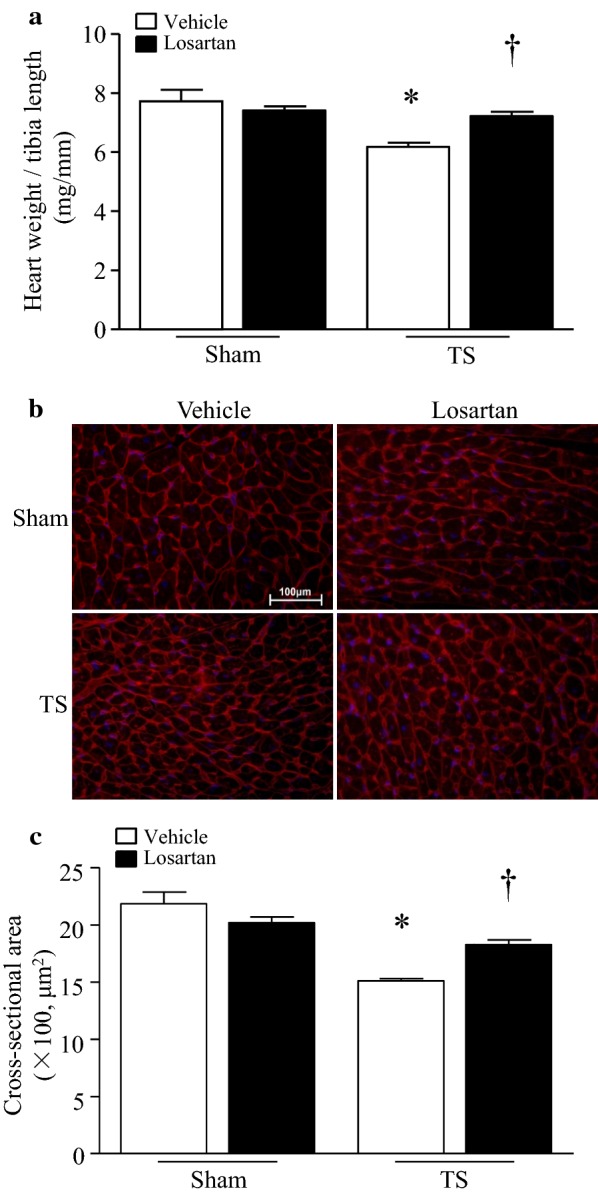
Administration of losartan preserves cardiomyocyte size and heart weight in tail-suspended mice. Tail-suspended mice were given losartan or vehicle in drinking water for 28 days. **a** The heart weight/tibia length ratio. **b** A representative staining of WGA in heart tissue sections. **c** Cardiomyocyte cross-sectional area. Data are mean ± SD, n = 6 in each group. *P < 0.05 vs. Sham + Vehicle and ^†^*P* < 0.05 vs. TS + Vehicle

Tail-suspension decreased heart weight and cardiomyocyte size (Fig. 3a–c), suggesting the occurrence of myocardial atrophy. The heart weight and cardiomyocyte size were much greater in losartan-treated compared with vehicle-treated mice after tail-suspension, suggesting that losartan may reduce myocardial atrophy in tail-suspended mice. In contrast, tail-suspension for 28 days did not increase caspase-3 activity in mouse hearts (Additional file 1: Figure S1), suggesting that apoptosis may be not involved in myocardial atrophy, which is consistent with a previous report [25].

### Losartan prevents oxidative stress in tail-suspended mouse hearts

**Fig. 4:**
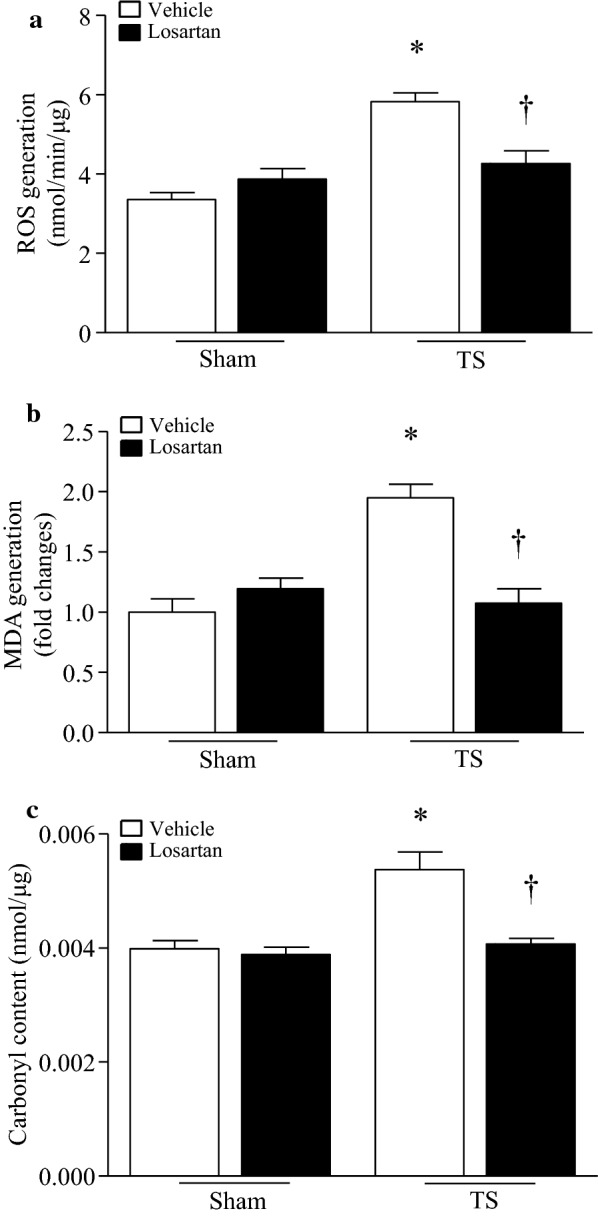
Losartan inhibits tail-suspension (TS)-induced oxidative stress in mouse hearts. Tail-suspended mice were given losartan or vehicle for 28 days. Oxidative stress was assessed by measuring ROS production (**a**), MDA production (**b**) and protein carbonyl content (**c**) in mouse hearts. Data are mean ± SD, n = 6 in each group. *P < 0.05 vs. Sham + Vehicle and ^†^*P* < 0.05 vs. TS + Vehicle

Angiotensin-II signaling has been implicated in promoting oxidative stress [10]. We therefore determined whether tail-suspension increased oxidative stress and whether blocking AT1R with losartan prevented tail-suspension-induced oxidative stress in the heart. As shown in Fig. 4a–c, tail-suspension induced a significant increase in ROS production, which correlated with oxidative damage as evidenced by increases in MDA production and protein carbonyl content. The ROS production and consequent oxidative damage were prevented by losartan treatment in tail-suspended mouse hearts. Thus, losartan prevented oxidative stress induced by tail-suspension.

### Administration of losartan inhibits NADPH oxidase activation in tail-suspended mouse hearts

**Fig. 5:**
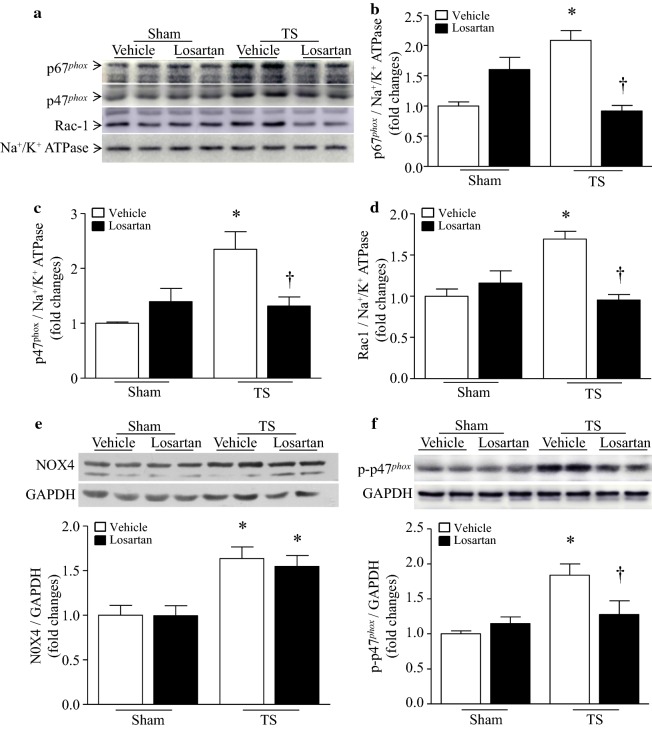
Losartan inhibits tail-suspension (TS)-induced NADPH oxidase activation in mouse hearts. Tail-suspended mice were given losartan or vehicle for 28 days. Cell membranes were isolated from mouse hearts and NADPH oxidase activation was determined by measuring p47^*phox*^, p67^*phox*^ and Rac1 in the membrane fractions relative to Na^+^/K^+^ ATPase. **a** Representative western blots from 2 out of 6 different hearts for p47^*phox*^, p67^*phox*^, Rac1 and Na^+^/K^+^ ATPase in cell membranes. **b**–**d** Quantitation for p67^*phox*^ (**b**), p47^*phox*^ (**c**) and Rac1 (**d**) relative to Na^+^/K^+^ ATPase. **e**, **f** The protein levels of NOX4 and phosphorylated p47 ^*phox*^ were determined in whole heart lysates by western blot analysis. Upper panels are representative western blots from 2 out of 6 different hearts for NOX4, phosphorylated p47^*phox*^ and GAPDH. Lower panels are quantitation for NOX4 and phosphorylated p47^*phox*^ relative to GAPDH. Data are mean ± SD, n = 6 in each group. *P < 0.05 vs. Sham + Vehicle and ^†^*P* < 0.05 vs. TS + Vehicle

Since NADPH oxidase including NOX2-containing NADPH oxidase and NOX4 is one of the major sources of ROS production in the heart [12, 13], we first determined whether tail-suspension-simulated microgravity induced NADPH oxidase activation. To this end, we analyzed the protein levels of cytosolic subunits of NOX2-containing NADPH oxidase including Rac1, p47^*phox*^ and p67^*phox*^ in the membrane fractions as activation of NOX2-containing NADPH oxidase requires the translocation of its cytosolic subunits to the membranes [14]. Tail-suspension resulted in increased protein levels of Rac1, p47^*phox*^ and p67^*phox*^ in cell membranes, indicative of NOX2-containing NADPH oxidase activation (Fig. 5a–d). We also determined the protein levels of NOX4 in whole heart tissue lysates as NOX4 is primarily regulated through transcriptional mechanisms [15]. Tail-suspension elevated the protein levels of NOX4 in mouse hearts (Fig. 5e, f). These results demonstrate that tail-suspension results in activation of NOX2-containing NADPH oxidase and NOX4 in the heart.

Administration of losartan reduced the protein levels of Rac1, p47^*phox*^ and p67^*phox*^ in cell membranes of tail-suspended mouse hearts (Fig. 5a–d), suggesting that blockage of AT1R prevents NOX2-containing NADPH oxidase activation. In contrast, losartan treatment did not change the protein levels of NOX4 in tail-suspended mouse hearts (Fig. 5e). Notably, these changes of NOX2-containing NADPH oxidase correlated with ROS production and oxidative damage (Fig. 4a–c). However, tail-suspension did not change SOD activity in mouse hearts and losartan had no significant effect on SOD activity in mouse hearts (Additional file 1: Figure S2). These results suggest that the inhibitory effect of losartan on ROS production may be mediated through prevention of NOX2-containing NADPH oxidase in tail-suspended mouse hearts.

To explore potential mechanisms by which losartan prevented NOX2-containing NADPH oxidase activation, we determined the levels of phosphorylated p47^*phox*^ in tail-suspended mouse hearts as p47^*phox*^ phosphorylation promotes the translocation of NOX2-containing NADPH oxidase cytosolic subunits to the membranes [14]. The levels of residual phosphorylation of Ser345 on p47^*phox*^ were much higher in tail-suspended mouse hearts relative to sham group (Fig. 5f). Interestingly, administration of losartan reduced the levels of residual phosphorylation of Ser345 on p47^*phox*^ (Fig. 5f), suggesting that blockage of AT1R prevents residual phosphorylation of Ser345 on p47^*phox*^ thereby inhibiting NOX2-containing NADPH oxidase activation in response to tail-suspension simulated microgravity.

**Fig. 6:**
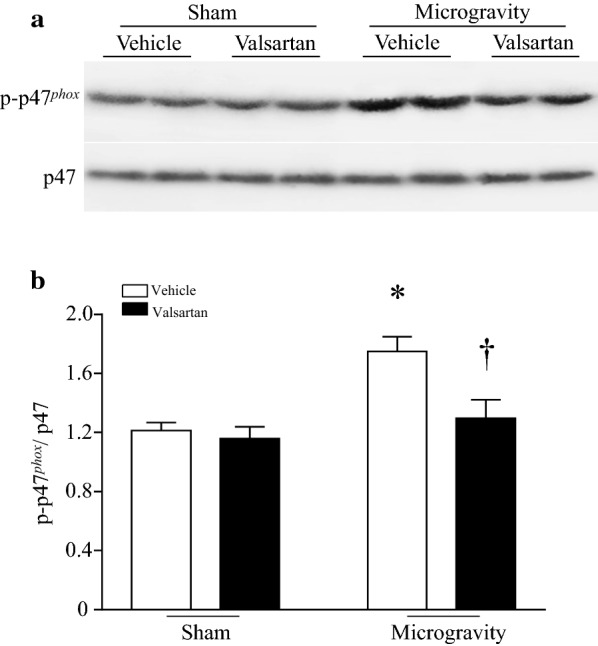
Effect of valsartan on p47^*phox*^ phosphorylation in cardiomyocytes. After isolation, neonatal mouse cardiomyocytes were cultured in normal media with valsartan (1 μM) or vehicle in the Rotary Cell Culture System to simulate microgravity. Twenty-four hours later, western blot was performed to analyze the protein levels of p47^*phox*^ (Ser345) in cardiomyocytes. **a** A representative western blot from 3 different cell cultures for phosphorylated p47^*phox*^ and total p47 ^*phox*^. **b** The quantitation for phosphorylated p47^*phox*^ relative to total p47 ^*phox*^. Data are mean ± SD, n = 3 in each group. *P < 0.05 vs. Sham + Vehicle and ^†^*P* < 0.05 vs. simulated microgravity + Vehicle

To provide further evidence to support the inhibitory effect of AT1R blockage on NADPH oxidase activation, we used another blocker of AT1R, valsartan (1 μM) in cultured neonatal cardiomyocytes under simulated microgravity. Microgravity increased the levels of residual phosphorylation of Ser345 on p47^*phox*^ in cardiomyocytes, indicative of NADPH oxidase activation. Incubation with valsartan prevented microgravity-induced increase in p47^*phox*^ phosphorylation (Fig. 6).

### Inhibition of NADPH oxidase preserves cardiomyocyte size and heart weight as well as myocardial function in tail-suspended mice

**Fig. 7:**
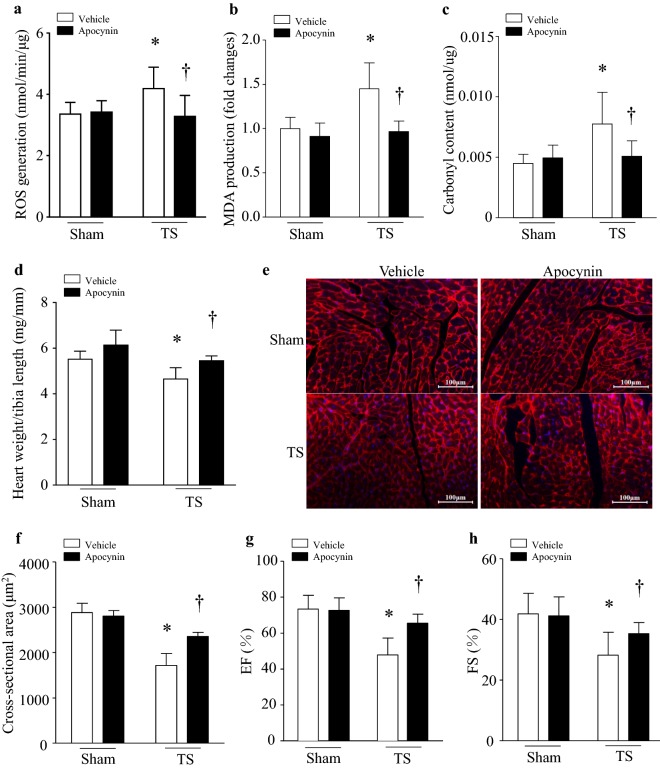
Inhibition of NADPH oxidase with apocynin prevents oxidative stress and myocardial abnormalities in tail-suspended mice. Tail-suspended mice were given apocynin or vehicle in drinking water for 28 days. Oxidative stress was assessed by measuring ROS production (**a**), MDA production (**b**) and protein carbonyl content (**c**) in mouse hearts. The heart weight/tibia length ratio (**d**) and cardiomyocyte cross-sectional area (**e**, **f**) were determined. **e** A representative staining of WGA in heart tissue sections. **f** Cardiomyocyte cross-sectional area. Myocardial dysfunction was assessed by ejection fraction (EF)% (**g**) and fraction shortening (FS)% (**h**). Data are mean ± SD, n = 6 in each group. *P < 0.05 vs. Sham + Vehicle and ^†^*P* < 0.05 vs. TS + Vehicle

To investigate the role of NADPH oxidase, we gave apocynin in drinking water to tail-suspended mice [20]. We chose apocynin because it is a well-established inhibitor of NADPH oxidase, which blocks the translocation of the cytosolic subunits p47^*phox*^ and p67^*phox*^ to the membrane fraction (a complex of NOX2 and p22^*phox*^) and thus inhibits NOX2-containing NADPH oxidase activation [26]. Administration of apocynin inhibited ROS production and prevented oxidative damage in tail-suspended mouse hearts (Fig. 7a–c). Tail-suspension decreased heart weight (Fig. 7d), cardiomyocyte size (Fig. 7e, f) and myocardial function in mice (Fig. 7g, h). Apocynin significantly increased cardiomyocyte size and heart weight, and preserved myocardial function in tail-suspended mice; however, it did not affect heart weight, cardiomyocyte size and myocardial function in mice without tail-suspension (Fig. 7d–g). These results support an important role of NADPH oxidase in myocardial abnormalities under microgravity.

### Losartan prevents MuRF1 expression in tail-suspended mouse hearts through unknown mechanisms independent of NADPH oxidase

**Fig. 8:**
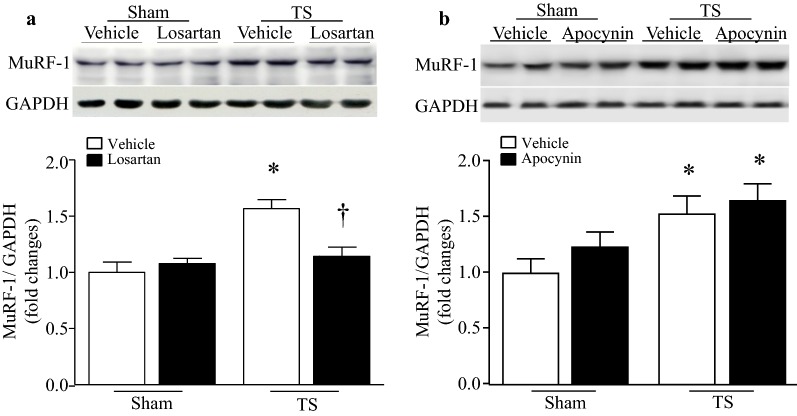
Losartan reduces whereas apocynin does not affect MuRF1 expression in tail-suspended mouse hearts. Tail-suspended mice were given losartan, apocynin or vehicle in drinking water for 28 days. Western blot analysis was performed to determine the protein levels of MuRF1 in heart tissue lysates (**a**, **b**). Upper panels are the representative western blots for MuRF1 protein expression from 2 out of 4–6 different hearts and lower panels are the quantification of MuRF1 protein relative to GAPDH. Data are mean ± SD, n = 4–6 in each group. *P < 0.05 vs. Sham + Vehicle and ^†^*P* < 0.05 vs. TS + Vehicle

MuRF1 induction has been demonstrated to be one of important mechanisms contributing to muscle atrophy [27]. We therefore determined the protein levels of MuRF1 in tail-suspended mouse hearts. Tail-suspension induced a significant increase in MuRF1 protein levels in vehicle-treated mice (Fig. 8), indicating the up-regulation of the ubiquitin–proteasome system. However, its levels were much lower in losartan-treated tail-suspended mice (Fig. 8a). In contrast, administration of apocynin did not change the protein levels of MuRF1 in tail-suspended mouse hearts (Fig. 8b). These results suggests that losartan reduces myocardial abnormalities at least partly by inhibiting MuRF1 dependent ubiquitin–proteasome system, and this inhibitory effect of losartan on MuRF1 expression is mediated through NADPH oxidase independent pathways.

## Discussion

The renin/angiotensin-II system is activated during human spaceflight [6] and head-down bed rest [7]. Consistently, our study demonstrates that the protein levels of angiotensin-II are elevated in the heart during tail-suspension simulated microgravity. It is known that the angiotensin-II system is counteracted by the apelin/apelin receptor system under physiological conditions [24]. In tail-suspended mouse hearts, we show that the apelin/apelin receptor system is down-regulated. These data indicate an imbalance between the angiotensin-II system and the apelin/apelin receptor system in tail-suspended mouse hearts with the former being increased and the latter decreased. The major finding of this study is that administration of losartan preserves cardiomyocyte size, heart weight and myocardial function in a mouse model of tail-suspension simulated microgravity. Losartan was also reported to prevent the development of myocardial atrophy in doxorubicin-injected mice [28]. Given that losartan is a well-known blocker of angiotensin-II receptor, our finding suggests that angiotensin-II signaling may promote myocardial atrophy under certain stress. On the contrary, angiotensin-II has been implicated in promoting myocardial hypertrophy under other pathological conditions [29]. The opposite functional effects of angiotensin-II (myocardial atrophy vs. hypertrophy) suggest that angiotensin-II’s actions may be dependent on pathological conditions.

Another important finding of this study is that NADPH oxidase is activated in tail-suspended mouse hearts. This is supported by the translocation of its cytosolic subunits including p47^*phox*^, p67^*phox*^ and Rac1 to cell membranes, up-regulation of NOX4 and concomitant oxidative stress. Our study further demonstrates that administration of losartan inhibits NOX2-containing NADPH oxidase activation and subsequent oxidative stress in tail-suspended mouse hearts. Although angiotensin-II has been implicated in promoting NADPH oxidase activation [10], we show for the first time that blockage of angiotensin-II receptor with losartan prevents residual phosphorylation of Ser345 on p47^*phox*^. The underlying molecular mechanisms remain unknown; however, previous studies have engaged the ERK1/2 and p38 signaling in mediating residual phosphorylation of Ser345 on p47^*phox*^ [30]. Indeed, activation of angiotensin-II receptor has been reported to promote ERK1/2 and p38 signaling [31, 32]. Nevertheless, further investigation is needed to determine whether the ERK1/2 and p38 signaling is operative in angiotensin-II-mediated residual phosphorylation of Ser345 on p47^*phox*^ during microgravity. On the contrary, losartan did not inhibit NOX4 expression in tail-suspended mouse hearts, suggesting that NOX4 induction is not mediated through the angiotensin-II signaling. Thus, the protective effects of losartan are not associated with NOX4 in tail-suspension simulated microgravity.

Oxidative stress is associated with the activation of protein degradation systems [33]. It is widely reported that prolonged muscle disuse results in an accumulation of oxidatively modified proteins and lipids, which may lead to the activation of the ubiquitin proteasome system [34, 35]. Under conditions of microgravity, it has been shown that oxidative stress is induced in the heart [36, 37]. This is also observed in the present study. In cardiomyocytes, several main sources of ROS production have been reported including NADPH oxidase, mitochondria, xanthine oxidase [38–40], etc. Notably, this study shows that NADPH oxidase is activated and oxidative stress is induced in mice with tail-suspension simulated microgravity, suggesting that NADPH oxidase activation may be an important mechanism contributing to oxidative stress and subsequent myocardial abnormalities in tail-suspended mice. This is indeed supported by our experimental evidence showing that inhibition of NADPH oxidase with apocynin preserved cardiomyocyte size, heart weight and myocardial function under a simulated condition of microgravity. Activation of NADPH oxidase has also been implicated in a variety of cardiac diseases [41].

In addition to inhibition of NADPH oxidase activation, losartan reduced tail-suspension-induced MuRF1 protein levels in the heart. MuRF1 is a well-known E3 ubiquitin ligase and it is increased transcriptionally in skeletal muscle during clinical conditions associated with skeletal muscle wasting [42]. Inhibition of MuRF1 attenuated skeletal muscle atrophy and dysfunction in cardiac cachexia [43]. Relevant to the present study, a recent report revealed that angiotensin-II induced skeletal muscle atrophy by increased MuRF1 expression [44]. Thus, the effect of losartan on preserving cardiomyocyte size and heart weight may be also mediated through MuRF1 induction during microgravity. Further evidence in support of our finding is that MuRF1 has been shown to inhibit cardiac hypertrophy in response to pressure overload through inhibition of the calcineurin-NFAT pathway [45].

The present study demonstrated that inhibition of NADPH oxidase with apocynin attenuated oxidative stress and increased cardiomyocyte size and heart weight in tail-suspended mice without affecting MuRF1 expression. This finding suggests that losartan prevents MuRF1 expression by unknown mechanisms independent of NADPH oxidase in tail-suspension simulated microgravity. This is somewhat unexpected as oxidative stress has been reported to induce MuRF1 expression [46]. Taken together, our data suggests that the protective effects of losartan may be mediated through MuRF1 dependent and independent mechanisms, and the MuRF1 independent mechanisms may include NADPH oxidase activation. It is worthwhile to mention that losartan has some off-target beneficial effects [47]. Currently, we do not know exactly how much cardiac protection of losartan results from its on-target or off-target effects in a mouse model of tail-suspension simulated microgravity. However, its inhibitory effect on NADPH oxidase activation most likely results from the blockage of angiotensin-II signaling as this pathway was reported to promote NADPH oxidase activation [10]. Additionally, another blocker of angiotensin-II receptor valsartan showed a similar inhibitory effect on NADPH oxidase activation under microgravity. Two different blockers of angiotensin-II receptor achieved a similar effect, strongly supporting that the protective effects of losartan may be mediated through blocking angiotensin-II signaling in microgravity-induced myocardial dysfunction. Future investigation is needed to clarify how losartan prevents MuRF1 expression in tail-suspended mouse hearts.

## Conclusion

Administration of losartan preserves cardiomyocyte size, heart weight and myocardial function in tail-suspended mice. These protective effects of losartan may be mediated through inhibition of NADPH oxidase activation and MuRF1 induction in microgravity. Thus, losartan may be a useful drug to prevent myocardial abnormalities during conditions of microgravity. Given that losartan as an effective blocker of angiotensin-II receptor has been widely used in clinical settings [48], the results from this study favor future translational research in astronauts using angiotensin-II receptor blockers.

## Supplementary information

**Additional file 1: Figure S1.** Effect of losartan on caspase-3 activity. Tail-suspended mice were given losartan or vehicle for 28 days. Caspase-3 activity was measured in heart tissue lysates. Data are mean ± SD, n = 6 in each group. **Figure S2.** Effect of losartan on SOD activity. Tail-suspended mice were given losartan or vehicle for 28 days. SOD activity was measured in heart tissue lysates. Data are mean ± SD, n =6 in each group.

## Data Availability

The datasets used and/or analyzed during the current study are available from the corresponding author on reasonable request. The mRNA expression profiles were deposited into the gene expression omnibus (GEO) database.

## Abbreviations

AT1R: angiotensin-II type 1 receptor
EF: ejection fraction
FS: fractional shortening
GAPDH: glyceraldehyde 3-phosphate dehydrogenase
MDA: malondialdehyde
MuRF1: ubiquitin ligase muscle RING-finger protein-1
ROS: reactive oxygen species
RT-PCR: reverse-transcription polymerase chain reaction
