# Supplementary material for: Administration of losartan preserves cardiomyocyte size and prevents myocardial dysfunction in tail-suspended mice by inhibiting p47phox phosphorylation, NADPH oxidase activation and MuRF1 expression
Source: J Transl Med. 2019 Aug 22;17:279. doi: 10.1186/s12967-019-2021-1 (PMC6704685; doi:10.1186/s12967-019-2021-1)
Supplement: Supplementary file 1 — Additional file 1: Figure S1. Effect of losartan on caspase-3 activity. Tail-suspended mice were given losartan or vehicle for 28 days. Caspase-3 activity was measured in heart tissue lysates. Data are mean ± SD, n = 6 in each group. Figure S2. Effect of losartan on SOD activity. Tail-suspended mice were given losartan or vehicle for 28 days. SOD activity was measured in heart tissue lysates. Data are mean ± SD, n =6 in each group. [file 12967_2019_2021_MOESM1_ESM.ppt]

## Slide 1
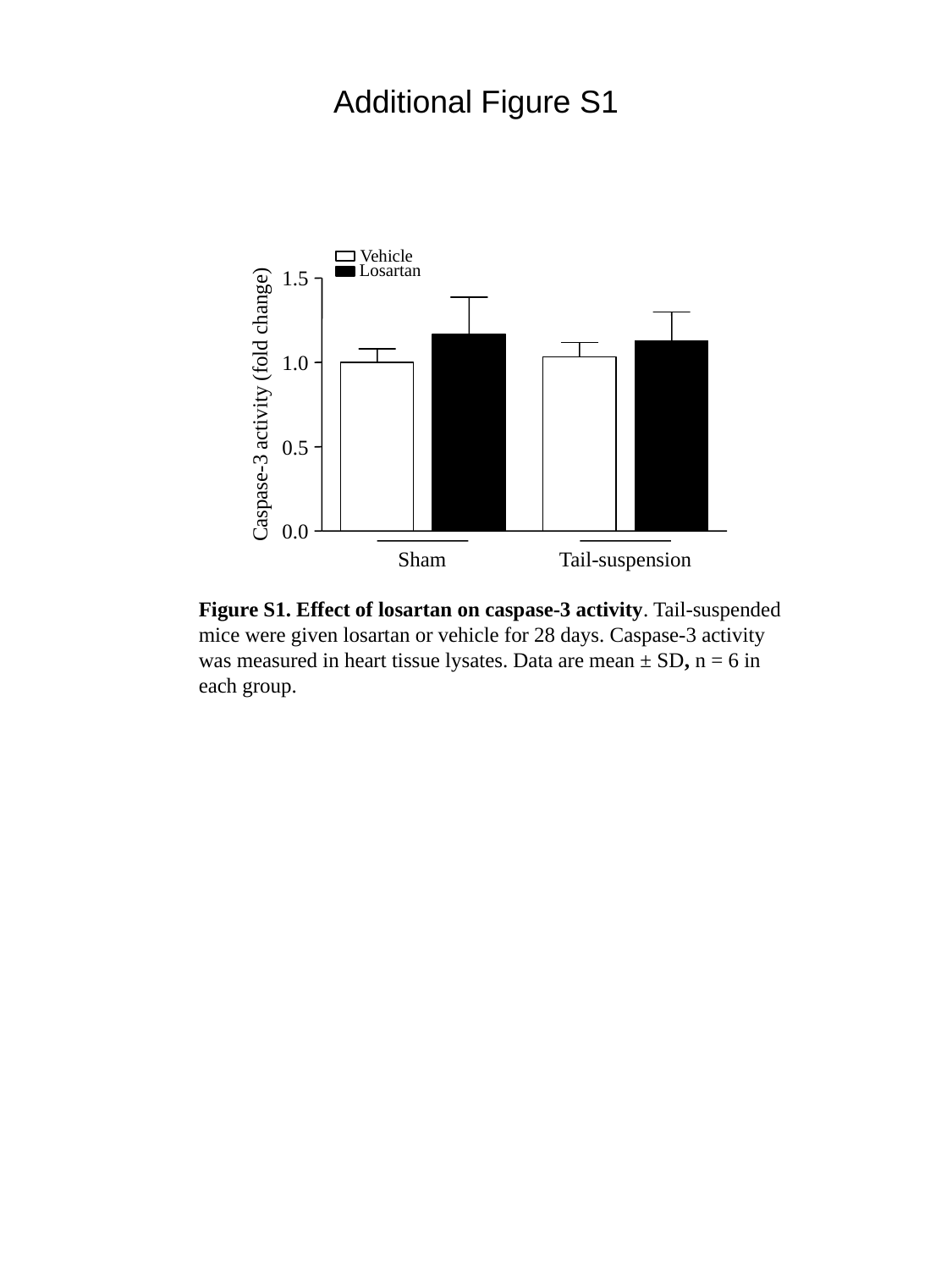

Additional Figure S1
Vehicle
Losartan
1.5
1.0
Caspase-3 activity (fold change)
0.5
0.0
Tail-suspension
Sham
Figure S1. Effect of losartan on caspase-3 activity. Tail-suspended mice were given losartan or vehicle for 28 days. Caspase-3 activity was measured in heart tissue lysates. Data are mean ± SD, n = 6 in each group.

## Slide 2
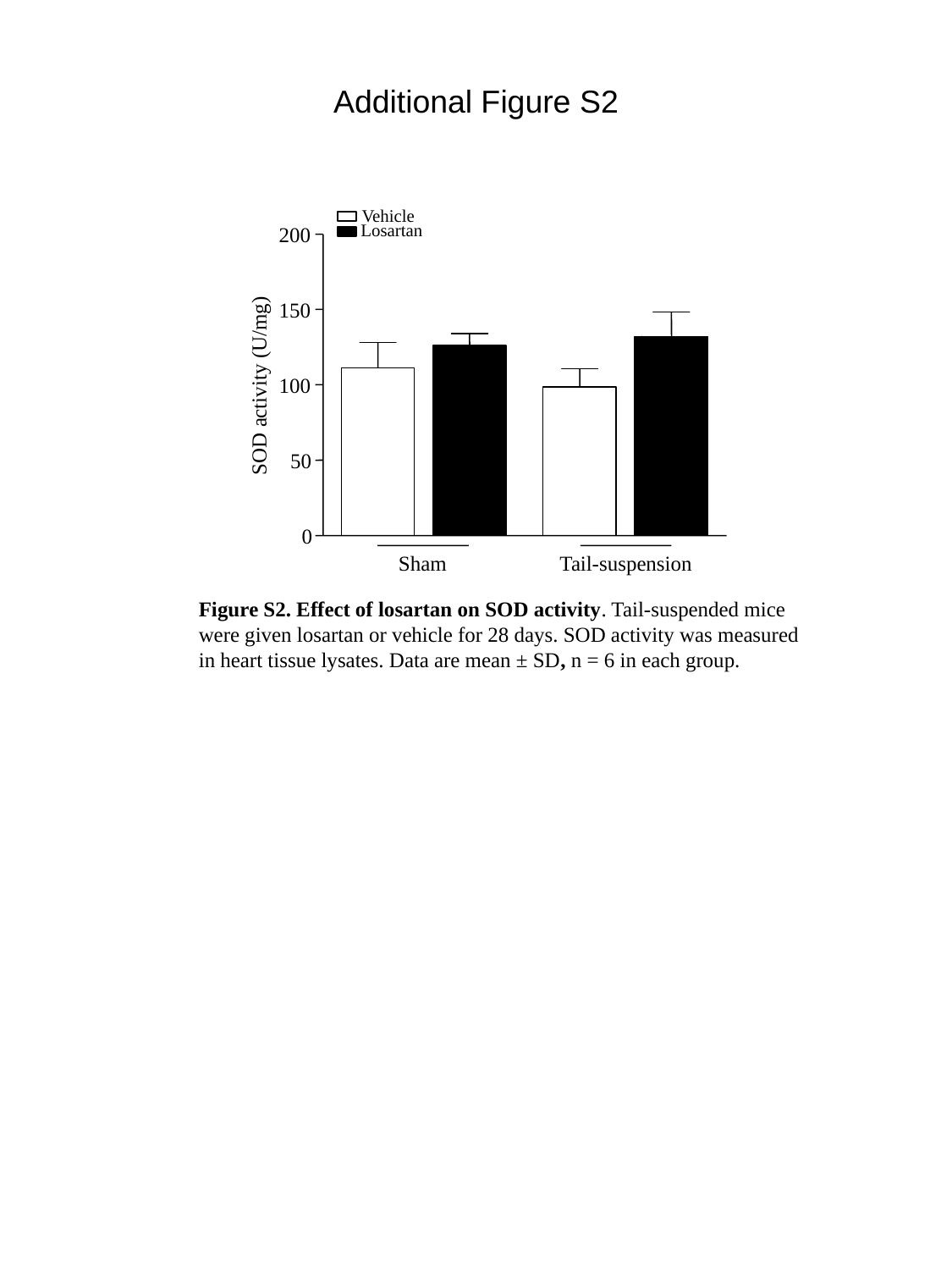

Additional Figure S2
Vehicle
Losartan
200
150
100
SOD activity (U/mg)
50
0
Sham
Tail-suspension
Figure S2. Effect of losartan on SOD activity. Tail-suspended mice were given losartan or vehicle for 28 days. SOD activity was measured in heart tissue lysates. Data are mean ± SD, n = 6 in each group.
